# The impact of COVID-19 on the dental hygienists: A cross-sectional study in the Lombardy first-wave outbreak

**DOI:** 10.1371/journal.pone.0262747

**Published:** 2022-02-02

**Authors:** Elena M. Varoni, Lucrezia Cinquanta, Marta Rigoni, Giulia Di Valentin, Giovanni Lodi, Paola Muti, Andrea Sardella, Antonio Carrassi

**Affiliations:** Dipartimento di Scienze Biomediche, Chirurgiche ed Odontoiatriche, Università degli Studi di Milano, Milan, Italy; China University of Mining and Technology, CHINA

## Abstract

The impact of COVID-19 on socio-economical activities has changed everyday life. Dental hygienists, who perform aerosol generating procedures, have been strongly affected by changes in routine procedures. This cross-sectional study aimed at carrying out an online survey among dental hygienists in Lombardy. The survey was implemented after the first-wave lockdown focusing on the level of knowledge on COVID-19 and Sars-CoV-2, the virus-related changes in their attitude and working routine, and the socio-economic effects. In this report, we included 313 questionnaires of respondents (259 Females, and 54 Males; age = 33 ± 9 years). A significant percentage of respondents acknowledged the use of “word of mouth” among colleagues (n = 114, 36%) and social networks (n = 113, 36%) to be up to date on COVID-19. About half of respondents correctly identified the main COVID-19 symptoms/signs, just 13% (n = 41) identified the routes of transmission. Three quarters of respondents (n = 234, 75%) were afraid of being infected during the clinical practice, and about half of them would be afraid to treat patients having symptoms attributable to COVID-19. Twenty-one percent (n = 67) of participants also thought about changing job. Air-polishing was identified as the highest risk procedure, and 82% (n = 256) reported that they eliminated its use. Most claimed they never had a swab or a serological test, with two respondents positive to molecular test (0.6%), and 12 positives to serological test (3.8%). More than half of the participants (65%; n = 202) complained the dental hygienist is not protected, despite a loss of earnings due to lockdown between 2,000 and 10,000 euros. This study demonstrated that dental hygienists were emotionally and economically affected by the pandemic, significantly changing their work routine. Anti-epidemic protocols are pivotal to react promptly and to contain the virus in the dental setting.

## Introduction

On 21st February 2020, the first confirmed Italian case of severe acute respiratory syndrome coronavirus 2 (SARS-CoV-2) infection, or coronavirus disease 2019 (COVID-19), was recorded in Codogno, Lombardy. Ever since, the number of infections increased dramatically up to more than 6,000 cases per day on the March 20^th^ [1], and Lombardy became the first outbreak in the world for the number of new cases and deaths, overcoming Wuhan City, China, where the COVID-19 pandemic began in December 2019. A two-month lockdown of the country successfully controlled the infection spreading. On May 4^th^, 2020, the Italian Government gradually re-opened the activities, including dental clinics, limited until that moment to the management of emergencies and cancer patients.

As the airborne is the leading route of transmission of the SARS-CoV-2, dental practitioners are at the highest risk of contagion during their work, particularly in case of dental aerosol-generating procedures, such as ultrasonic scalers, air polishing or high‐speed rotor use [2,3]. Along these lines, the dental hygienist (DH) is the professional role at the highest threat of contagion, according to a “COVID-19 score” based on the following three items: contact with others, physical proximity, exposure to disease and infection [4]. This picture can produce enormous distress at the work, and, consistent with the other medical personnel, may show controversial feelings about covering the role of healthcare provider and, in the meantime, be a family member (e.g., spouse/parents), with an inner conflict between one’s professional responsibility and the fear of infecting relatives [5,6].

As for many other working activities, the dental team has been asked to revolutionize the way of performing the routine activities [7], as occurred during HIV epidemic in the nineties, when universal precautions where advocated to reduce at minimum the risk of transmission [8]. DHs, who primarily carry out aerosol-generating procedures such as dental scaling [2,4,9–11], have been particularly involved in this revolution due to COVID-19. Indeed, during these procedures, DHs are recommended to wear specific personal protective equipment (PPE), including filtering face piece type 2 mask (FFP2, also named N95) or equivalent or higher-level respirator, such as disposable filtering facepiece respirator, or elastomeric respirator, as well as eye protection (goggles or a face shield that covers the front and sides of the face), gloves, and a gown [7,12,13]. Procedures that generate aerosol should ideally take place in an airborne infection isolation room, and should be followed by careful disinfection and cleaning [7]. In this perspective, operating times increase, along with the stress and fatigue of the dental operator.

To the best of our knowledge, the literature has particularly focused on dentists [6,14–20], but evidence is still scant on the socio-economic and emotional impact of the first wave of the COVID-19 pandemic on dental hygienists. In addition, the level of knowledge of Sars-CoV-2 and the source of information used by the dental hygienist to keep updated has not been deeply explored. The present cross-sectional questionnaire-based study aimed to investigate the impact that the COVID-19 epidemic had on the DH’s professional profile and activity, in particular in the Lombardy Region (Northern Italy), after the first wave of COVID-19. The survey analysed DH’s level of knowledge about COVID-19 and Sars-CoV-2; the changes the virus has made in their attitude and the way they carry out their working routine; and the financial protection they received from government.

## Materials and methods

### Study design and participants

A cross-sectional study was carried out by means of an online survey sent to all DHs working in Lombardy and affiliated to the Lombardy Institutional Boards of DHs (459 males, 1097 females; mean age: 34 ± 9 years). DHs working in Lombardy, were identified via the Lombardy Institutional Boards.

Sample size calculation was performed using an online tool (https://www.idsurvey.com/en/sample-size-calculator/) setting the following parameters: margin of error = 5%, confidence interval (CI) = 95% and response distribution = 50%. This calculation was based on the total population under investigation (i.e., Lombardy DHs, n = 1556), the minimum number of required respondents was 309.

The survey was conducted from June 9th, 2020, up to July 15^th^, 2020.

Ethical approval for this questionnaire-based study was obtained from the University of Milan Ethics Committee, in accordance with the Declaration of Helsinki. The informed consent about privacy and data management was included at the beginning of the questionnaire and thus obtained by all participants.

#### Inclusion criteria

Questionnaires completed in full by graduated dental hygienists, affiliated to Lombardy Institutional Boards, were included.

#### Exclusion criteria

We excluded questionnaires in case a participant has answered more than once (considering as correct the first one given by the individual), or in case the respondent was not graduated as a dental hygienist, or in case the respondent was not a dental hygienist affiliated to Lombardy Institutional Boards, or in case of missing information/answers in the form.

### Questionnaire and procedure

An online tool was used to conduct the survey (https://docs.google.com/). For granting pseudonymization, a code identifier was given to each questionnaire. The participants were approached using social media, dedicated mailing lists, and forums.

The questionnaire was prepared according to previous literature [21,22]. A preliminary questionnaire was pre-tested on a small group of dental hygienists and dentists (n = 6); all the items were discussed by the authors and modified accordingly. The results of this preliminary survey were not included in the main study. Besides demographic data, the questionnaire (Table 1) showed three main domains, i.e. the level of knowledge about COVID-19 [COVID-19 symptoms and signs, and Sars-CoV-2 ways of transmission according to previous literature [23–25]; the impact on their professional activity, including changes in the working routine and attitude (as the fear of treating patients with suspected symptoms/signs of COVID-19), and epidemiological data about COVID-19 infection; and the financial aid received from the government.

**Table 1:** Study questionnaire administered to dental hygienists (translated from Italian).

**Demographic data**
Age (years):
Gender:
Select the Lombard province in which you carry out your profession: • Bergamo • Brescia • Como • Cremona • Lecco • Lodi • Mantova • Milano • Monza Brianza • Pavia • Sondrio • Varese
He is domiciled and / or works in one or more outbreak provinces (Bergamo, Brescia, Cremona, Lodi)*? Yes / No * *Outbreaks of the first COVID-19 wave* If so, please specify which one
Do you work as (more than one reply is allowed): • Activities with the national health system • Owner of a private clinic • Unemployed • Employee at a private facility • Self-employed • Other:…
**Knowledge about Sars-CoV-2**
How do you find information about the SARS-CoV-2 virus? (more than one reply is allowed) • Scientific journals • Newspaper/ news broadcasts • Word of mouth among colleagues • Biomedical Databases / Search Engines (e.g., PubMed) • Institutional websites (e.g., WHO, Istituto superior di Sanità, Ministry of Health, etc. . .) • Social Networks (Facebook, Twitter, Instagram) • I don’t go looking for information • Other: …
Can you identify the main signs and symptoms of COVID-19? (more than one reply is allowed) • Fever • Dry cough • Fatty cough • Vomit • Asthenia/muscular weakness • I do not know • Other: …
An asymptomatic person could be contagious? Yes / No / I do not know
Can you identify the main ways of transmission of the virus SARS-CoV-2? (more than one reply is allowed) • Blood • Saliva • Droplets • Mucus • Direct contact with intact skin • Direct contact with mucosae • Conjunctival secretions • I do not know
**Impact of COVID-19 on the dental hygienist’s professional activity**
Do you have or have been afraid of getting infected during clinical practice? Yes / No
From now on, will you be more afraid of treating patients with symptoms attributable to COVID-19? Yes / No
Given the spread of COVID-19, have you thought about changing kind of job? Yes / No
Will you avoid going to work from now on if you have a fever / flu-like symptoms? Yes / No
Given the spread of COVID-19, what PPE are you using? (more than one reply is allowed) • Gloves • Face shield • Safety goggles • Clogs shoes • Disposable protective shoes • Protective cap • Disposable gown • Surgical mask • FFP2 mask • FFP3 mask • Disposable filtering facepiece respirator • Disposable protective cap • Disposable sleeves • Double pair of gloves • Other:…
Under what circumstances do you use FFP2 / FFP3 masks? • Always, also for follow-up visit, periodontal charting and bleaching procedure • For procedures that generate aerosols only • Never
Who provided you with the PPE you use? • They have been provided to me free of charge in all the clinics I collaborate with • I had to buy them at my own expense in all the clinics in which I collaborate • They were provided to me for free in some clinics, while in others I had to buy them at my own expense • Other:..
Are there any procedures that you believe may expose you to a greater risk of contagion? (more than one reply is allowed) • Scaling • Polishing • Air-polishing • Manual scaling and root planing • Sealants • Bleaching • Laser therapy • Periodontal probing/charting • Other:…
Given the spread of COVID-19, which mouth rinse does the patient perform before starting the operating procedures?? (more than one reply is allowed) • 0.2% chlorhexidine-based mouthwash • Mouthwash based on essential oils • 1% hydrogen peroxide solution • Solution with povidone iodine 0.2% • Water and bicarbonate • I avoid rinsing the patient • I ask the patient for a double rinse: first with a solution with povidone iodine 0.2% or hydrogen peroxide 1% or cetylpyridinium chloride 0.05% -0.1% and then one with chlorhexidine 0.2% • Other:…
How has it changed your way of working? (more than one reply is allowed) • I use only manual instruments (scaler, curettes. . .) • I have decreased / eliminated the use of powders (air-polishing) • I have reduced / eliminated the use of sonic and / or ultrasonic instruments • I haven’t changed the way I work • Other:…
If you are a collaborator, have you been given the opportunity to manage your agenda? • Yes, in all the clinics in which I collaborate • Yes, in some of the clinics I work with • No
Before the spread of SARS-CoV-2, how many patients did you treat on average in a day? • <3 • 3–4 • 5–6 • 7 –8 • >8
After the spread of SARS-CoV-2, how many patients do you treat on average in a day? • <3 • 3–4 • 5–6 • 7–8 • >8
Have you ever received a rhino-pharyngeal swab for the molecular diagnosis of SARS-CoV-2 infection? • Yes, I was positive but asymptomatic • Yes, I was positive symptomatic • Yes, I was negative • I wanted to have the swab because I have symptoms, but I couldn’t • No, I’ve never had a swab • Rather not answer
Did you perform the serological test for the diagnosis of COVID-19? • Yes, I was positive for IgM • Yes, I was positive for IgG • Yes, I was positive for IgM and IgG • Yes, I was positive, but the test did not distinguish between IgG and IgM • Yes, but I was negative • No, I did not take the serological test • Rather not answer
**Government financial aid**
Do you believe that the category of dental hygienists is protected from a health and / or financial point of view? • Yes, always • Yes, but only from a health point of view • Yes, but only from a financial point of view • Never
Estimate your LOSS of income due to the forced closure of dental practices: • ≤ 1000 euros • 1000–2000 euros • 2000–5000 euros • 5000–10000 euros • ≥ 10000 euros • I am unemployed • I do not know • Other:…
Have you applied for financial subsidies (e.g., Istituto Nazionale di Previdenza Sociale–INPS)? Yes / No
The price of your procedures: • It has remained unchanged in all the clinics I collaborate with • It has increased in all the clinics I work with • It has decreased in all the clinics where I work • In some clinics where I work it has increased, while in others it has remained unchanged • Other:…

### Statistical analysis

Statistical analysis was performed using Stata software, version 13.1. Continuous variables were described as mean ± standard deviation (SD). Dichotomous variables, including number of replies to questionnaire’s questions, were expressed as percentages and compared using Pearson’s χ^2^ or Fisher’s exact test, as appropriate. To find correlation among different variables (questionnaire answers), Spearman’s correlation was performed. Statistical significance was set at P ≤ .05.

The study was conformed to S1 Checklist (STROBE Supplementary Information).

## Results

Three hundred and fifty-three questionnaires were collected (55 males; 259 females). Thirty-nine of them were filled more than once by the same respondent by mistake, in one case, the respondent was a student; therefore 40 questionnaires were excluded. Three hundred and thirteen questionnaires were finally included for further analysis. Table 2 summarizes the demographic data of study participants (54 males, 259 females; mean age 33 ± 9 years). The median responders’ age was 31 years (1^st^ quartile: 27; 3^th^ quartile: 37), thus half of the responders were from 27 to 37 years old, indicating a broad range of specialists with an average work experience. Moreover, the 95.85% of the responders were self-employed (freelancers), highlighting a certain uniformity of the professional qualification.

**Table 2:** Socio-demographic data of study participants.

Socio-demographic variables	Number of respondents
**Total, n**	313
Male, n (%)	54 (17.2)
Female, n (%)	259 (82.8)
Age, mean (sd), years	33 (9)
**Working place (provinces of Lombardy)** *	**Number of answers** ^ψ^
Bergamo, n (%)	41 (9.7)
Brescia, n (%)	37 (8.8)
Como, n (%)	31 (7.3)
Cremona, n (%)	20 (4.7)
Lecco, n (%)	10 (2.4)
Lodi, n (%)	16 (3.8)
Mantova, n (%)	6 (1.4)
Monza Brianza, n (%)	36 (8.5)
Milan, n (%)	151 (35.9)
Pavia, n (%)	11 (2.6)
Sondrio, n (%)	11 (2.6)
Varese, n (%)	52 (12.3)
Outbreak provinces*	**Number of answers** ^ψ ψ^
Bergamo, n (%)	40 (9.5)
Brescia, n (%)	36 (8.5)
Cremona, n (%)	20 (4.7)
Lodi, n (%)	18 (4.3)
**Working position**	313
Activities with the national health system, n (%)	7 (2.1)
Owner of a private clinic, n (%)	5 (1.5)
Unemployed, n (%)	2 (0.6)
Employee at a private facility, n (%)	16 (4.8)
Self-employed, n (%)	300 (90.7)
Partner of a private practice, n (%)	1 (0.3)

n = number; sd = standard deviation

* = more than one answer was possible

^ψ^ based on 313 professionals who gave 422 answers

^ψ ψ^ based on 91 professionals (27%) who gave 114 answers.

### Knowledge about COVID-19

Overall, only a few of the study participants (n = 20, 6.4%) used reliable scientific sources for finding information about COVID-19, i.e., declaring the use of at least one among scientific journals, biomedical databases, institutional websites, medical board or CME courses.

**Fig 1:**
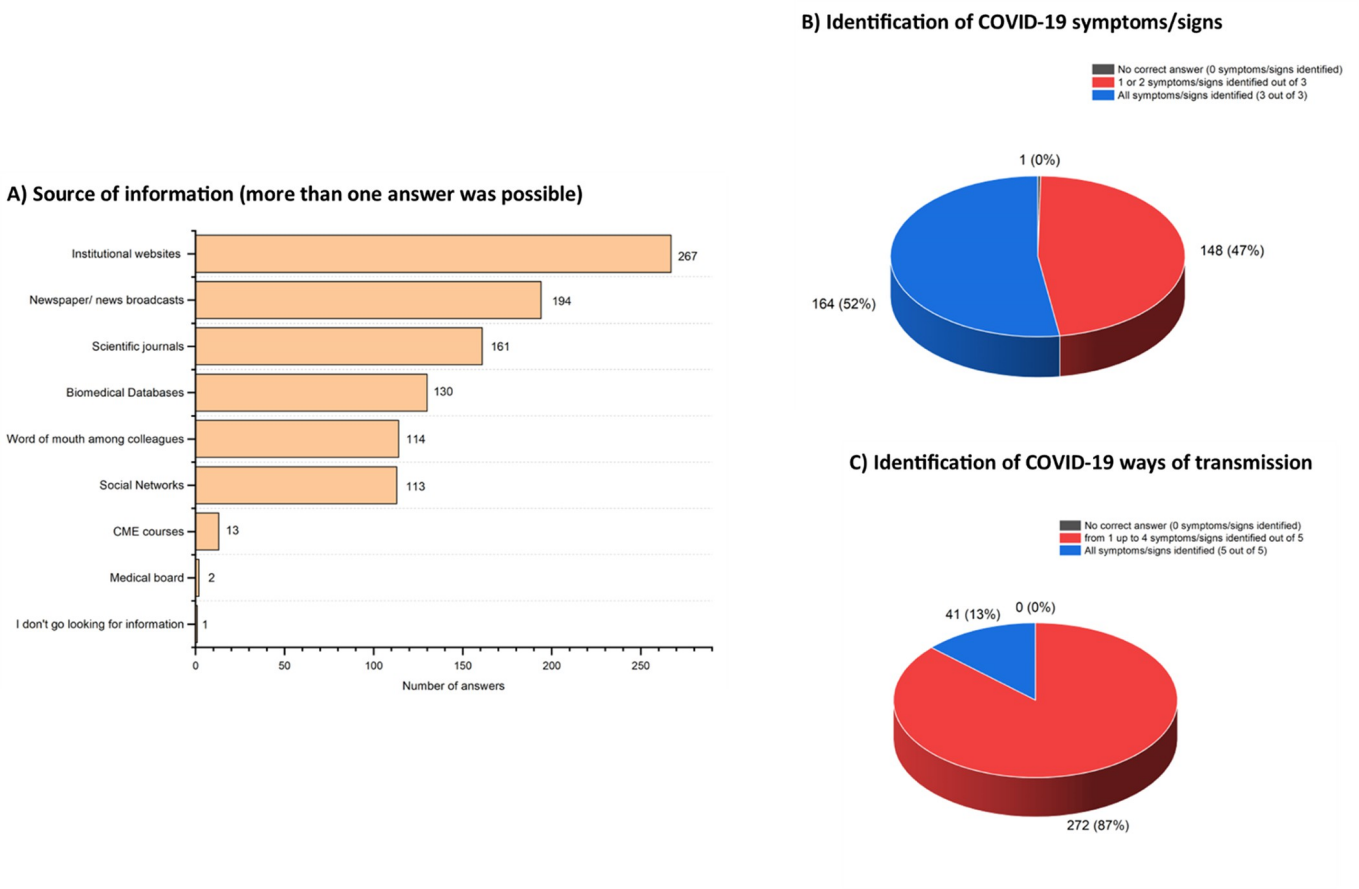
A) Sources used by study participants to find information about COVID-19. B) Knowledge of study participants about the three main COVID-19-related symptoms/signs (fever, dry cough, asthenia/muscular weakness) ([23–25]). C) Knowledge of study participants about the five main COVID-19-related ways of transmission (saliva, mucus, droplets, conjunctival secretions and direct contact with mucosa).

The most consulted source of information were institutional websites (e.g., WHO, Istituto Superiore di Sanità, Ministry of Health) (n = 267, 85.3%). A significant percentage of respondents also acknowledged the use of word of mouth among colleagues (n = 114, 36.4%) and social networks (e.g., Twitter, Facebook) (n = 113, 36.1%) (Fig 1A).

Most of the participants recognized that an asymptomatic patient could be contagious (n = 301, 96.2%). Almost half of the respondents correctly identified the three main symptoms/signs (fever, dry cough, asthenia/muscular weakness), while the other half recognized only one or two of them (Fig 1B) (S1 Table), without a significant correlation with the reliability of sources consulted (Spearman’s correlation coefficient = 0.01, P = .80) or the provenience of the outbreak from the provinces (Spearman’s correlation coefficient< -0.01, P = .89).

Similarly, only the 13.1% (n = 41) identified all the possible ways of transmission, i.e., saliva, mucus, droplets, conjunctival secretions and direct contact with mucosa, while the others identified from 1 to 4 ways (Fig 1C) (S2 Table), without a significant correlation with the reliability of sources consulted (Spearman’s correlation coefficient = -0.06, P = .27) or the provenience of the outbreak from the provinces (Spearman’s correlation coefficient< 0.01, P = .98).

#### Impact on the DH’s professional activity

**Fig 2:**
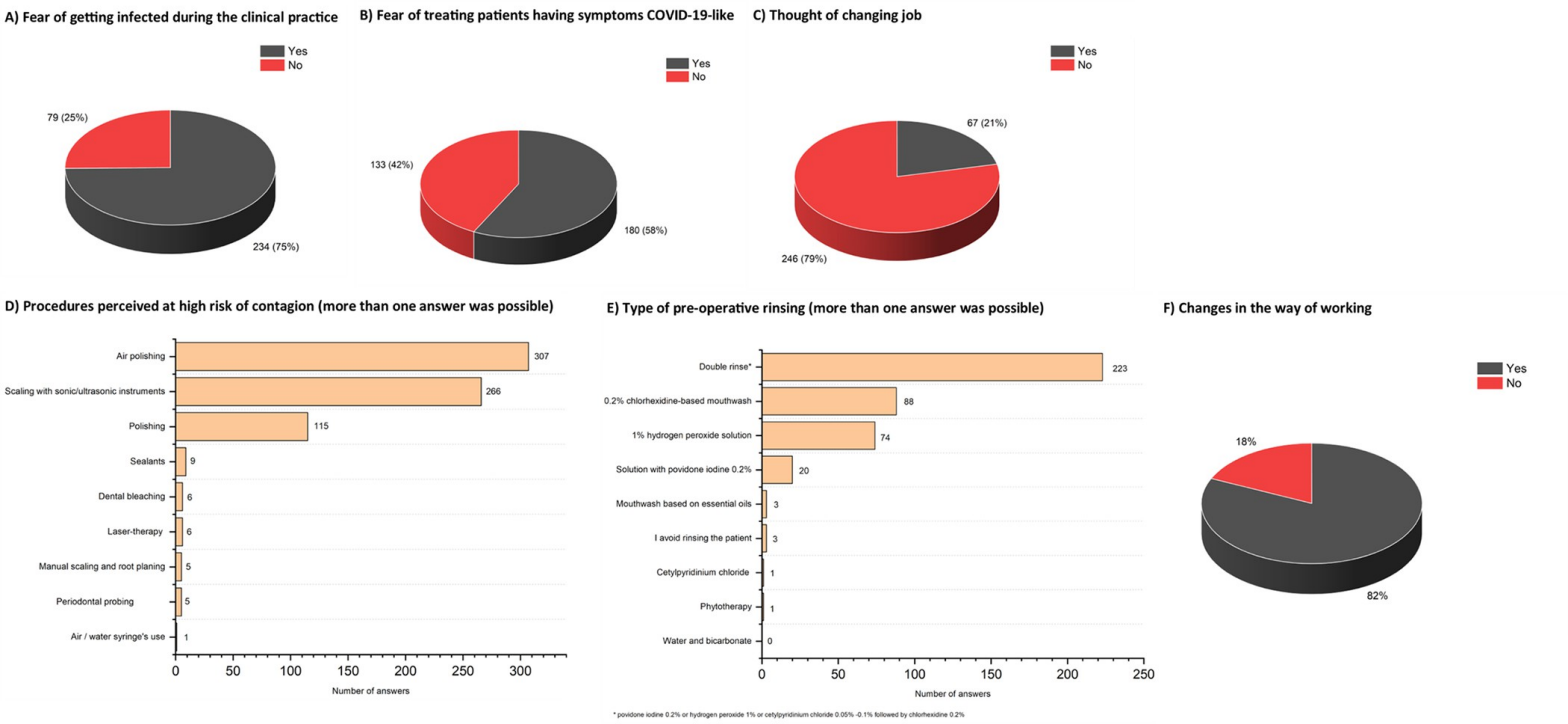
A) Answers of study participants to the question: “Are you or were you afraid of getting infected during clinical practice?”. B) Answers of study participants to the question: “From now on, will you be more afraid of treating patients with symptoms attributable to COVID-19?”. C) Answers of study participants to the question: “Due to the spreading of COVID-19, have you thought about changing kind of job?”. D) Procedures that may expose the DH to a greater risk of contagion, as perceived by study participants (more than one reply was allowed). E) Type of mouth rinse that the patient is asked to perform before starting the operating procedures, as reported by study participants (more than one reply was allowed). F) Number of respondents who reported a change in the way of working.

Most of the study participants were afraid of becoming infected during clinical practice (n = 234, 75%) (Fig 2A), and would avoid going to work in case of fever/flu-like symptoms (n = 288, 92%). About half of them (n = 180, 58%) would be afraid of treating patients having symptoms attributable to COVID-19, and 21% (n = 67) of participants also thought about changing their job (Fig 2B and 2C). Overall, no correlation could be found with the provenience of the outbreak from the provinces (Spearman’s correlation coefficients = -0.01, -0.05, -0.02, P = .99, .40, .65, respectively).

The most used PPE, such as gloves and face shield, were mainly supplied by the employer free of charge (S3 Table). FFP2 mask was used for all procedures by the majority of study participants (n = 258, 82.4%), while 16.9% of the responders (n = 53) used them only for procedures that generate aerosol- and only 0.7% (n = 2) declared they never use this item.

Among the procedures perceived to have the highest risk of contagion, the study participants reported air-polishing (n = 307, 98.1%) (Fig 2D). In most of cases (n = 223, 71.2%), a pre-operative double rinse was performed (Fig 2E).

Consistently, the majority reported changing their way of working (n = 256, 82%) (Fig 2F), in particular by eliminating the use of powders and air-polishing (n = 244, 78%) (S4 Table).

Although only 16% (n = 50) of the study participants could not manage the agenda according to the changes of the working activities, most of them significantly reduced the number of patients treated during the day, compared to the pre-pandemic activity (*P <* .*01*, Fisher’s exact test) (S5 Table).

**Fig 3:**
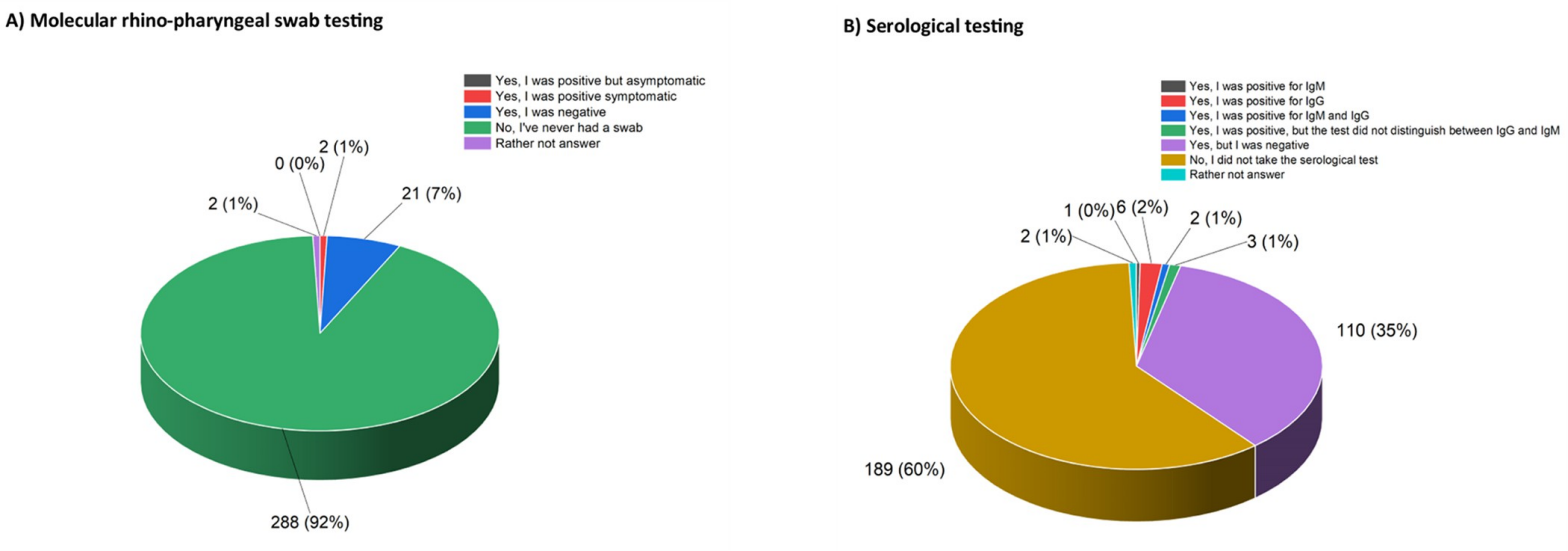
Epidemiological data about respondents’ results to A) molecular Sars-CoV-2 test, and B) serological IgG/IgM Sars-CoV-2 test.

About epidemiological data, most claimed they never had a swab or a serological test (Fig 3A and 3B), with a significant higher proportion of individuals who received the molecular test and resulted negative within the group of study participants coming from one of the first-wave outbreak provinces (P < .01, Fisher’s exact test; Spearman’s correlation coefficient = 0.17, P < .01). Only two respondents were positive to molecular test (0.6%), while only 12 were positive to IgG and/or IGM serological test (3.8%).

### Government financial support

**Fig 4:**
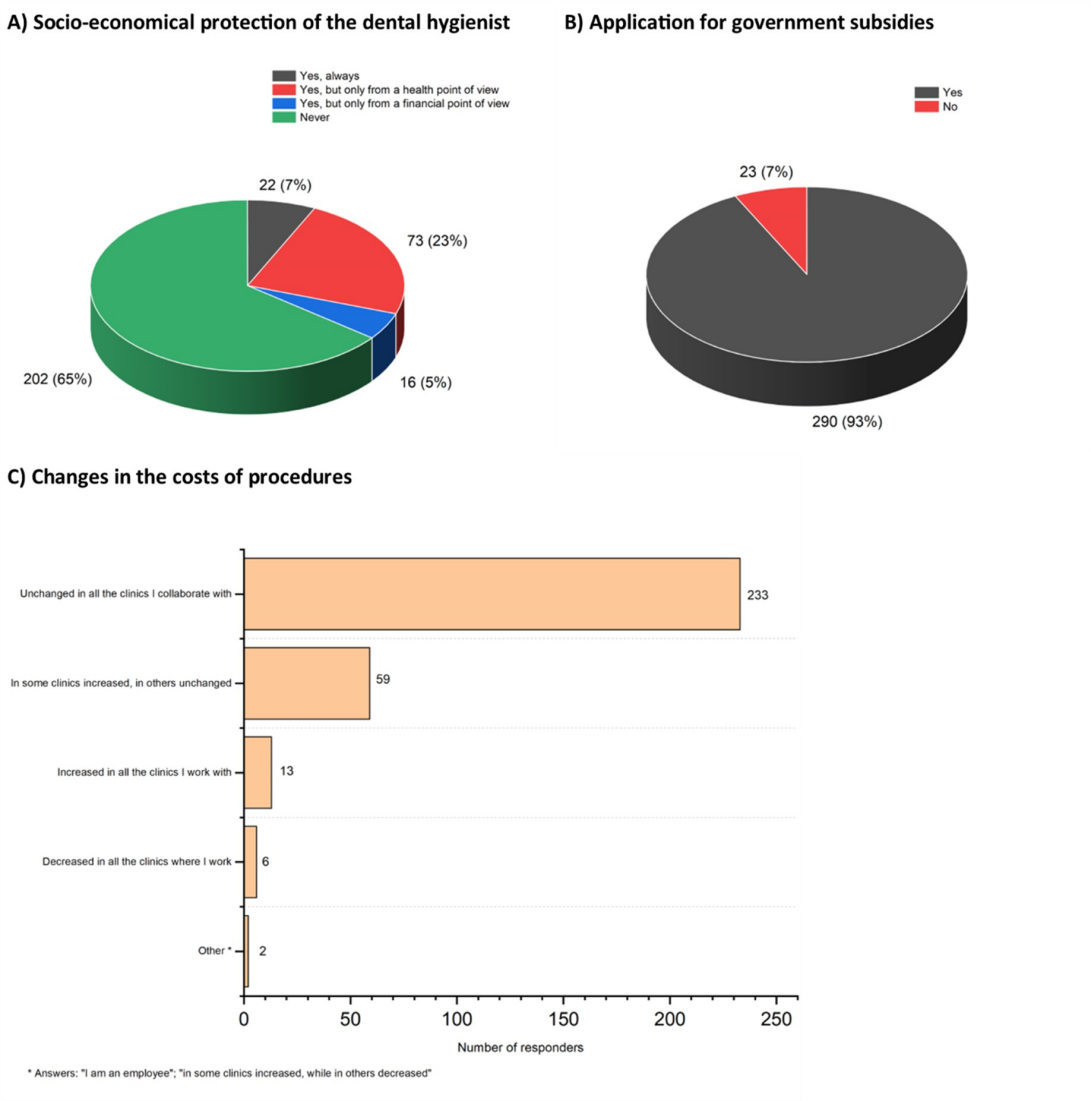
A) Answers of study participants to the question: “Do you believe that the category of DHs is protected from a health and / or financial point of view?”. B) Number of respondents who applied for financial support from the government. C) Number of respondents who reported changes in the price of procedure due to COVID-19 pandemic.

In terms of socio-economical protection, 65% (n = 202) of the study participants reported that the DH is never protected, neither from a financial point of view nor from a health point of view. However, almost all of them (n = 290, 93%) applied for financial subsidies (es. Istituto Nazionale di Previdenza Sociale–INPS) (Fig 4A and 4B).

Overall, the extent of the loss of earnings due to the lockdown was between 2,000 and 5,000 € for 43% (n = 135) of the respondents, and between 5,000 and 10,000 € for 30% (n = 96) (S6 Table).

At re-opening, the prices of procedures predominantly remained unchanged in the clinics (Fig 4C).

## Discussion

COVID-19 pandemic has led to an enormous change in the way healthcare professionals conduct their working routine, as happened in the 1980s when current PPE and universal safety procedures were introduced in dentistry as a response to HIV epidemic [26]. The same procedures and safety devices allowed to contain, within the dental setting, subsequent epidemics, such as SARS, HCV and, nowadays, COVID-19, as our study also found, reporting a low percentage of positive individuals. Dentists and DHs, indeed, are used to employ PPE during their routine practice, and are specifically trained during their academic course. This reinforces the importance of setting specific anti-epidemic protocols, to be implemented in case of future epidemics, and which should always be based on universal safety procedures and PPE.

Although many studies in the literature have focused, since the beginning of the pandemic, on the dentists’ knowledge levels, attitudes and awareness regarding Covid-19 [6,14–20], the professional figure of the DH has been neglected, despite the high impact of the pandemic on DHs. To the best of our knowledge, data on DH knowledge of the disease, changes in their working routine and psyco-emotional attitudes, as well as epidemiological data and financial impact, are still scanty. This study aims to elucidate all these aspects and to investigate the effect of the COVID-19 pandemic on this professional role, at higher risk of contagion [4]. A better understanding of these issues would reinforce the role of PPE in the fight against the pandemic, and the need for adequate safety procedures.

A high response rate was obtained here, and study participants could be considered an appropriate representative sample of the entire population (DHs working in Lombardy). The mean age of the sample (33 ± 9 years) corresponded to the mean age of the population of interest (34 ± 9 years); a similar percentage of female/male ratio was found, with 82.7% of women and 17.2% of men among respondents, while 70.5% of women and 29.5% of men in the population of interest. Moreover, the study participants came from the various provinces Lombardy, with an adequate distribution, proportional to the number of DHs operating in each of them.

From this study it emerges that, although the most consulted source of information were institutional websites (85.3%), a non-negligible percentage of respondents declared that they use newspapers, news broadcasts, “word of mouth” among colleagues and social networks to keep up-to-date, similarly to the cross-sectional survey on dentists in Lebanon [17]. These could provide information that may be scientifically unreliable, although the use of social media for sharing knowledge in healthcare and keeping up to date with the literature has been recently advocated [27,28]. However, about half of the respondents correctly identified the three main COVID-19 symptoms/signs, and only 13% (n = 41) identified the main routes of transmission. This emphasizes the need of better defining the postgraduate training and academic education for the consultation of biomedical sources. Moreover, the figures described here are not consistent with those reported among dentists, coming from different populations, who appeared with a satisfactory knowledge level of the disease [17,19, 20, 29].

From the psyco-emotional point of view, 75% of the respondents were afraid of getting infected during the clinical practice, and about half of them would be afraid of treating patients having symptoms attributable to COVID-19, which is consistent with data from other reports on dentists [14,15,17,20,30], including those from Lombardy [16]. Even, 21% (n = 67) of study participants also considered changing job. A recent survey highlighted that the pandemic produced an 8% reduction in DH employment, in most of cases voluntary, due to concerns on COVID-19 and family issues [31]. Literature supports high psychological distress, anxiety and depression among dental staff, including DHs, especially during the time of the outbreak [6,18,32] and particularly related to the increased level of uncertainty surrounding the COVID-19 pandemic and oral health care of the patient, and towards personal life and infectivity [33].

The most used PPE were disposable gloves, face shield, disposable gown, FFP2 mask, followed by safety goggles, clogs shoes, and protective cap. Most of the study participants (82.4%), in particular, used FFP2 mask, for all procedures. A previous cross-sectional survey on Italian DHs [34] found that the most frequently employed PPE were protective glasses or visor, disposable gloves and surgical mask. The difference in the frequency of PPE, mainly of FFP2 mask, could be ascribed to the different prevalence between Lombardy and the rest of Italy, during the first-wave of COVID-19: the high prevalence area could have led to the more frequent use of PPE implemented, consistent with the data reported on Lombard dentists [16].

In this study, air-polishing was identified as the highest risk procedure, followed by ultrasonic/sonic scaling, and 82% of respondents declared they significantly changed their way of working, in 72% of cases by eliminating its use. These findings are consistent with the international recommendations to reduce the need of air-polishing during COVID-19 outbreak [7].

On what concerns the epidemiological data on COVID-19 among Lombard DHs, most claimed they never had a swab or a serological test, with two respondents positive for the molecular test (0.6%), and 12 positives for the serological test (3.8%). This is probably due to the lack of availability and access to testing during the first-wave of COVID-19, in this high prevalence area. Overall, the data support a low rate of contagion among dental staff, as found in other cross-sectional surveys on Lombard dentists [16], reporting 0.25% positivity to Sars-CoV-2, and on Italian DHs (0.9%) [34], as well as found in cross-sectional studies including data from Portugal (0.5%), Spain (1.4%-1.6%), China (0.8%) and US (0.9%) [32,35]. The universal guidelines for the control of infection, adopted in dentistry during the AIDS pandemic and lasted to present day, might have overcome the spread of this respiratory disease in dental settings [26,32].

Despite being at high risk, 65% complained the DH is a non-protected professional category, neither from a health nor from a financial point of view. A loss of earnings due to the lockdown was mostly between the 2,000–10,000 €. Economic, ethical, social and professional concerns have been reported among dental staff during COVID-19 outbreak [36], such concerns may be protracted in the long term, as safety protocols, especially for procedures producing aerosols (dressing/undressing, dental room ventilation), reduced the number of patients treated in one day. Coping strategies, including procedures implemented for patient management and infection control, adoption of appropriate policies and new technologies related to tele-dentistry should alleviate these anxieties.

From a methodological point of view, a high response rate was obtained, and study participants could be considered an appropriate sample representative of the entire population (DHs working in Lombardy). The mean age of the sample (33 ± 9 years) corresponded to the mean age of the population of interest (34 ± 9 years); there is a similar percentage of female/male ratio, with 82.7% of women and 17.2% of men among respondents, while 70.5% of women and 29.5% of men in the population of interest. Moreover, the study participants came from the various Lombard provinces, with adequate distribution, which was proportional to the number of DHs working in each of them.

Limitations of this study include its cross-sectional design, which precludes causal inferences and also is prone to selection bias, since we used an online survey and not all DHs could have had quick access to emails and social networks. Furthermore, these findings should be taken into consideration given that the study was conducted at the beginning of the pandemic: the period in which the questionnaire was sent referred to the reopening of activities after the first lockdown, when there was great uncertainty and the level of knowledge regarding COVID-19 was still in its infancy. Nowadays, vaccine availability and better understanding of the virus, COVID-19 therapies and routine safety protocols are likely to have changed the perception of healthcare professionals, including DHs, for the better. However, these findings may be useful in the event of a new health emergency linked to pandemics, allowing for a timelier health and socio-economical management of the situation. A further limitation is related to study population, which refers to DHs working in one region of Italy, Lombardy, although, at that time, it was the outbreak.

In conclusion, this cross-sectional study provided an accurate picture of the impact of the first-wave of COVID-19 on the DH working in Lombardy, area an of high prevalence. Not in all cases the source of information on COVID-19 was scientifically adequate, referring to social networks and word of mouth among colleagues. The majority reported the fear to be infected during clinical practice, even thinking about changing job. The most hazardous procedures were identified as air-polishing and scaling with sonic/ultrasonic instruments, and most of the respondents avoided these working practices. The economic toll paid by this professional figure was important, in the face of a socio-economic protection perceived as lacking. The infection rate, although biased by the very high percentage of untested individuals, appeared low, with only two respondents positive for Sars-CoV-2. The dental team, including DHs, is usually well-trained to manage the risk of cross-contamination and epidemic infection in the work routine and this emphasizes, once again, the role of preventive protocols to contain potential future pandemics.

## Supporting information

S1 Checklist STROBE statement—checklist of items that should be included in reports of *cross-sectional studies*. (DOC)

S1 Table Answers to the item: “Can you identify the main signs and symptoms of COVID-19? (more than one reply is allowed)”. (DOCX)

S2 Table Answers to the item: “Can you identify the main ways of transmission of the virus SARS-CoV-2? (more than one reply is allowed)”. (DOCX)

S3 Table Answers to the items: “Given the spread of COVID-19, what PPE are you using? (more than one reply is allowed)” and “who provided you those PPE?”. (DOCX)

S4 Table Answers to the item: “How has it changed your way of working? (more than one reply is allowed)”. (DOCX)

S5 Table Answers to the items: “If you are a collaborator, have you been given the opportunity to manage your agenda?”; “Before the spread of SARS-CoV-2, how many patients did you treat on average in a day?”; “After the spread of SARS-CoV-2, how many patients did you treat on average in a day?”. (DOCX)

S6 Table Answers to the items: “Estimate your loss of income due to the forced closure of dental practices”. (DOCX)
