# Supplementary material for: The impact of COVID-19 on the dental hygienists: A cross-sectional study in the Lombardy first-wave outbreak
Source: PLoS One. 2022 Feb 2;17(2):e0262747. doi: 10.1371/journal.pone.0262747 (PMC8809622; doi:10.1371/journal.pone.0262747)
Supplement: S1 Checklist — (DOC) [file pone.0262747.s001.doc]

STROBE Statement—Checklist of items that should be included in reports of ***cross-sectional studies***

|  | Item No | Recommendation |
| --- | --- | --- |
| **Title and abstract** | 1 | (*a*) Indicate the study’s design with a commonly used term in the title or the abstract done, see title |
|  |  | (*b*) Provide in the abstract an informative and balanced summary of what was done and what was found done, see title |
| Introduction | | |
| Background/rationale | 2 | Explain the scientific background and rationale for the investigation being reported done, see introduction |
| Objectives | 3 | State specific objectives, including any prespecified hypotheses done, see introduction |
| Methods | | |
| Study design | 4 | Present key elements of study design early in the paper done, see MM |
| Setting | 5 | Describe the setting, locations, and relevant dates, including periods of recruitment, exposure, follow-up, and data collection done, see MM |
| Participants | 6 | (*a*) Give the eligibility criteria, and the sources and methods of selection of participants done, see MM |
| Variables | 7 | Clearly define all outcomes, exposures, predictors, potential confounders, and effect modifiers. Give diagnostic criteria, if applicable done, see MM |
| Data sources/ measurement | 8* | For each variable of interest, give sources of data and details of methods of assessment (measurement). Describe comparability of assessment methods if there is more than one group NA |
| Bias | 9 | Describe any efforts to address potential sources of bias done, see MM |
| Study size | 10 | Explain how the study size was arrived at done, see MM |
| Quantitative variables | 11 | Explain how quantitative variables were handled in the analyses. If applicable, describe which groupings were chosen and why done, see MM |
| Statistical methods | 12 | (*a*) Describe all statistical methods, including those used to control for confounding done, see MM |
|  |  | (*b*) Describe any methods used to examine subgroups and interactions done, see MM |
|  |  | (*c*) Explain how missing data were addressed NA |
|  |  | (*d*) If applicable, describe analytical methods taking account of sampling strategy NA |
|  |  | (*e*) Describe any sensitivity analyses NA |
| Results | | |
| Participants | 13* | (a) Report numbers of individuals at each stage of study—eg numbers potentially eligible, examined for eligibility, confirmed eligible, included in the study, completing follow-up, and analysed done, see results |
|  |  | (b) Give reasons for non-participation at each stage NA |
|  |  | (c) Consider use of a flow diagram NA |
| Descriptive data | 14* | (a) Give characteristics of study participants (eg demographic, clinical, social) and information on exposures and potential confounders done, see results |
|  |  | (b) Indicate number of participants with missing data for each variable of interest done, see results |
| Outcome data | 15* | Report numbers of outcome events or summary measures done, see results |
| Main results | 16 | (*a*) Give unadjusted estimates and, if applicable, confounder-adjusted estimates and their precision (eg, 95% confidence interval). Make clear which confounders were adjusted for and why they were included done, see results |
|  |  | (*b*) Report category boundaries when continuous variables were categorized done, see results |
|  |  | (*c*) If relevant, consider translating estimates of relative risk into absolute risk for a meaningful time period NA |
| Other analyses | 17 | Report other analyses done—eg analyses of subgroups and interactions, and sensitivity analyses done, see results |
| Discussion | | |
| Key results | 18 | Summarise key results with reference to study objectives done, see Discussion |
| Limitations | 19 | Discuss limitations of the study, taking into account sources of potential bias or imprecision. Discuss both direction and magnitude of any potential bias done, see Discussion |
| Interpretation | 20 | Give a cautious overall interpretation of results considering objectives, limitations, multiplicity of analyses, results from similar studies, and other relevant evidence done, see Discussion |
| Generalisability | 21 | Discuss the generalisability (external validity) of the study results done, see Discussion |
| Other information | | |
| Funding | 22 | Give the source of funding and the role of the funders for the present study and, if applicable, for the original study on which the present article is based Done, No funding received |

*Give information separately for exposed and unexposed groups.

**Note:** An Explanation and Elaboration article discusses each checklist item and gives methodological background and published examples of transparent reporting. The STROBE checklist is best used in conjunction with this article (freely available on the Web sites of PLoS Medicine at http://www.plosmedicine.org/, Annals of Internal Medicine at http://www.annals.org/, and Epidemiology at http://www.epidem.com/). Information on the STROBE Initiative is available at www.strobe-statement.org.
