# Supplementary material for: The impact of COVID-19 on the dental hygienists: A cross-sectional study in the Lombardy first-wave outbreak
Source: PLoS One. 2022 Feb 2;17(2):e0262747. doi: 10.1371/journal.pone.0262747 (PMC8809622; doi:10.1371/journal.pone.0262747)
Supplement: S1 Table — (DOCX) [file pone.0262747.s002.docx]

**Supporting Information**

**S1 Table. Answers to the item: “Can you identify the main signs and symptoms of COVID-19? (more than one reply is allowed)”**

| **Symptoms/Signs** | **Number of respondents** |
| --- | --- |
| Fever, n (%) | 311 (99.4) |
| Dry cough, n (%) | 253 (80.8) |
| Fat cough, n (%) | 18 (5.75) |
| Vomit, n (%) | 39 (12.5) |
| Asthenia/muscular weakness, n (%) | 257 (82.1) |
| I do not know, n (%) | 0 (0.0) |
| Ageusia, n (%) | 27 (8.6) |
| Anosmia, n (%) | 21 (6.7) |
| Headache, n (%) | 3 (1.0) |
| Diarrhoea, n (%) | 11 (3.5) |
| Sore throat, n (%) | 2 (0.6) |
| Conjunctivitis, n (%) | 7 (2.2) |
| Dyspnoea, n (%) | 5 (1.6) |
| Hearing impairment, n (%) | 1 (0.3) |
| Lack of appetite, n (%) | 1 (0.3) |
| Nasal congestion, n (%) | 4 (1.3) |
|  |  |
