# Supplementary material for: The impact of COVID-19 on the dental hygienists: A cross-sectional study in the Lombardy first-wave outbreak
Source: PLoS One. 2022 Feb 2;17(2):e0262747. doi: 10.1371/journal.pone.0262747 (PMC8809622; doi:10.1371/journal.pone.0262747)
Supplement: S2 Table — (DOCX) [file pone.0262747.s003.docx]

**S2 Table. Answers to the item: “can you identify the main ways of transmission of the virus SARS-CoV-2? (more than one reply is allowed)”**

| **Ways of transmission** | **Number of respondents** |
| --- | --- |
| Blood, n (%) | 34 (10.9) |
| Saliva, n (%) | 290 (92.65) |
| Droplets, n (%) | 294 (93.9) |
| Mucus, n (%) | 164 (52.4) |
| Direct contact with intact skin, n (%) | 19 (6.1) |
| Direct contact with mucosae, n (%) | 125 (39.9) |
| Conjunctival secretions, n (%) | 182 (58.15) |
| I do not know, n (%) | 0 (0.0) |
| Other, n (%) | 0 (0.0) |
