# Supplementary material for: The impact of COVID-19 on the dental hygienists: A cross-sectional study in the Lombardy first-wave outbreak
Source: PLoS One. 2022 Feb 2;17(2):e0262747. doi: 10.1371/journal.pone.0262747 (PMC8809622; doi:10.1371/journal.pone.0262747)
Supplement: S3 Table — (DOCX) [file pone.0262747.s004.docx]

**S3 Table. Answers to the items: “given the spread of COVID-19, what PPE are you using? (more than one reply is allowed)” and “who provided you those PPE?”**

| **Type of PPE** | **Number of respondents** |
| --- | --- |
| Gloves, n (%) | 293 (93.31) |
| Face shield, n (%) | 305 (97.1) |
| Safety goggles, n (%) | 240 (76.4) |
| Clogs shoes, n (%) | 243 (77.4) |
| Disposable protective shoes, n (%) | 117 (37.3) |
| Protective cap, n (%) | 280 (89.2) |
| Disposable gown, n (%) | 307 (97.8) |
| Surgical mask, n (%) | 273 (86.9) |
| FFP2 mask, n (%) | 287 (91.4) |
| FFP3 mask, n (%) | 50 (15.9) |
| Disposable filtering facepiece respirator, n (%) | 53 (16.9) |
| Disposable protective cap, n (%) | 267 (85.0) |
| Disposable sleeves, n (%) | 50 (15.9) |
| Double pair of gloves, n (%) | 164 (52.2) |
| Water repellent suit, n (%) | 4 (1.3) |
| Electric respirator, n (%) | 3 (1.0) |
| Aerosol aspirator, n (%) | 1 (0.3) |
| Ozone, n (%) | 1 (0.3) |
| **How the PPE were obtained:** |  |
| They have been provided to me free of charge in all the clinics I collaborate with, n (%) | 199 (63.6) |
| I had to buy them at my own expense in all the clinics in which I collaborate, n (%) | 7 (2.2) |
| They were provided to me for free in some clinics, while in others I had to buy them at my own expense, n (%) | 102 (32.6) |
| They were provided to me for free in all clinics, but I made personal purchases of my own choice, n (%) | 4 (1.3) |
| I bought them for everyone since the clinic was mine, n (%) | 1 (0.3) |
