# Supplementary material for: The impact of COVID-19 on the dental hygienists: A cross-sectional study in the Lombardy first-wave outbreak
Source: PLoS One. 2022 Feb 2;17(2):e0262747. doi: 10.1371/journal.pone.0262747 (PMC8809622; doi:10.1371/journal.pone.0262747)
Supplement: S4 Table — (DOCX) [file pone.0262747.s005.docx]

**S4 Table. Answers to the item: “How has it changed your way of working? (more than one reply is allowed)”**

| **Change in way of working** | **Number of respondents** |
| --- | --- |
| I have decreased / eliminated the use of powders (air-polishing), n (%) | 244 (78%) |
| I haven't changed the way I work, n (%) | 57 (18.2) |
| I use only manual instruments (scaler, curettes ...), n (%) | 22 (7.0) |
| I have reduced / eliminated the use of sonic and / or ultrasonic instruments, n (%) | 68 (21.7) |
| I avoid using the air / water syringe, n (%) | 1 (0.3) |
| I constantly change the air in the room, n (%) | 1 (0.3) |
| I use the double aspirator | 4 (1.3) |
