# Supplementary material for: The impact of COVID-19 on the dental hygienists: A cross-sectional study in the Lombardy first-wave outbreak
Source: PLoS One. 2022 Feb 2;17(2):e0262747. doi: 10.1371/journal.pone.0262747 (PMC8809622; doi:10.1371/journal.pone.0262747)
Supplement: S5 Table — (DOCX) [file pone.0262747.s006.docx]

**S5 Table. Answers to the items: “If you are a collaborator, have you been given the opportunity to manage your agenda?”; “Before the spread of SARS-CoV-2, how many patients did you treat on average in a day?”; “After the spread of SARS-CoV-2, how many patients did you treat on average in a day?”**

| **Possibility to manage the agenda** | **Number of respondents** |
| --- | --- |
| No, n (%) | 50 (16.0) |
| Yes, in all the clinics, n (%) | 175 (55.9) |
| Yes, in some of the clinics, n (%) | 82 (26.2) |
| No answer, n (%) | 6 (1.9) |
| **Patient per day (before pandemic)*** |  |
| <3, n (%) | 0 (0.0) |
| 3-4, n (%) | 0 (0.0) |
| 5-6, n (%) | 19 (6.1) |
| 7-8, n (%) | 101 (32.3) |
| >8, n (%) | 193 (61.7) |
| **Patient per day (after pandemic)*** |  |
| <3, n (%) | 2 (0.7) |
| 3-4, n (%) | 22 (7.0) |
| 5-6, n (%) | 124 (39.6) |
| 7-8, n (%) | 123 (39.3) |
| >8, n (%) | 42 (13.4) |

Legend: *= *P < .01*, Fisher’s exact test between before and after pandemic
