# Supplementary material for: The impact of COVID-19 on the dental hygienists: A cross-sectional study in the Lombardy first-wave outbreak
Source: PLoS One. 2022 Feb 2;17(2):e0262747. doi: 10.1371/journal.pone.0262747 (PMC8809622; doi:10.1371/journal.pone.0262747)
Supplement: S6 Table — (DOCX) [file pone.0262747.s007.docx]

**S6 Table. Answers to the items: “Estimate your loss of income due to the forced closure of dental practices”**

| **Loss of earnings estimate** | **Number of respondents** |
| --- | --- |
| <1000 euros, n (%) | 9 (2.9) |
| 1000-2000 euros, n (%) | 33 (10.4) |
| 2000-5000 euros, n (%) | 135 (43.1) |
| 5000-10000 euros, n (%) | 96 (30.7) |
| >10000 euros, n (%) | 16 (5.1) |
| I do not know, n (%) | 17 (5.4) |
| I am unemployed, n (%) | 4 (1.3) |
| I took vacation, n (%) | 1 (0.3) |
| I was in quarantine, n (%) | 1 (0.3) |
| I had a reduction of -80%, n (%) | 1 (0.3) |
